# Upstream Regulators of Fibroblast Growth Factor 23

**DOI:** 10.3389/fendo.2021.588096

**Published:** 2021-02-26

**Authors:** Danielle M. A. Ratsma, M. Carola Zillikens, Bram C. J. van der Eerden

**Affiliations:** Laboratory for Calcium and Bone Metabolism and Erasmus MC Bone Centre, Department of Internal Medicine, Erasmus University Medical Center, Rotterdam, Netherlands

**Keywords:** FGF23, osteocytes, phosphate, vitamin D, iron deficiency, hypoxia, inflammation, erythropoiesis

## Abstract

Fibroblast growth factor 23 (FGF23) has been described as an important regulator of mineral homeostasis, but has lately also been linked to iron deficiency, inflammation, and erythropoiesis. FGF23 is essential for the maintenance of phosphate homeostasis in the body and activating mutations in the gene itself or inactivating mutations in its upstream regulators can result in severe chronic hypophosphatemia, where an unbalanced mineral homeostasis often leads to rickets in children and osteomalacia in adults. FGF23 can be regulated by changes in transcriptional activity or by changes at the post-translational level. The balance between O-glycosylation and phosphorylation is an important determinant of how much active intact or inactive cleaved FGF23 will be released in the circulation. In the past years, it has become evident that iron deficiency and inflammation regulate FGF23 in a way that is not associated with its classical role in mineral metabolism. These conditions will not only result in an upregulation of *FGF23* transcription, but also in increased cleavage, leaving the levels of active intact FGF23 unchanged. The exact mechanisms behind and function of this process are still unclear. However, a deeper understanding of FGF23 regulation in both the classical and non-classical way is important to develop better treatment options for diseases associated with disturbed FGF23 biology. In this review, we describe how the currently known upstream regulators of FGF23 change *FGF23* transcription and affect its post-translational modifications at the molecular level.

## Introduction

In 1959 a first mention was made by Andrea Prader of a circulating “rachitonic” substance that was the cause of tumor-induced osteomalacia (TIO). He described a case of an 11-year-old girl, who suddenly developed severe rickets. A tumor was identified between her ribs, and resection resulted in curation of her rickets (1, 2). The first evidence of a circulating phosphaturic factor comes from experiments in *hyp* mice, a model for X-linked hypophosphatemic rickets (XLH), in 1989, where it was shown that a circulating factor in *hyp* mice could induce hypophosphatemia in wild-type mice (3). This concept was already suggested a year earlier, when a resected tumor from a TIO patient caused hypophosphatemia when transplanted into a nude mouse (4). This phosphaturic factor was first identified as fibroblast growth factor 23 (FGF23) in 2000 by the autosomal-dominant hypophosphatemic rickets (ADHR) consortium when mutations in FGF23 where found to be causing ADHR (5). Besides FGF23, other phosphatonins such as FGF7, secreted-frizzled related protein (sFRP4) and matrix extracellular phosphoglycoprotein (MEPE), have been identified but descriptions of these factors are beyond the scope of this review (6).

FGF23 is predominantly produced by osteocytes and osteoblasts in the skeleton and has a key function in regulating phosphate homeostasis (7). When serum phosphate levels rise due to bone resorption or absorption of dietary phosphate, FGF23 levels increase. FGF23 can bind to the fibroblast growth factor receptor 1 (FGFR1) in the kidneys, but needs its co-receptor, α-klotho, to execute its functions (8). Binding of FGF23 in the kidney has several effects. First of all, the expression of type II sodium-phosphate cotransporter NaPi-2a (encoded by *SLC34A1*), present in the cell surface of the renal proximal tubules is downregulated, resulting in decreased phosphate reabsorption (9). Secondly, FGF23 inhibits 1α-hydroxylase in the kidney, the key enzyme for the conversion of inactive 25-hydroxyvitamin D (25(OH)D) to the active 1,25-dihydroxyvitamin D_3_ (1,25(OH)_2_D_3_) (10). Reduced levels of 1,25(OH)_2_D_3_ result in lower expression of NaPi-2b (*SLC34A2*) in the small intestine, and therefore less absorption of dietary phosphate (11, 12). The parathyroid gland is another important target for FGF23, where it is able to downregulate parathyroid hormone (PTH) synthesis and secretion in a α-klotho-dependent manner (13, 14). PTH has an important role in regulating renal phosphate reabsorption as it downregulates NaPi-2a/c (*SLC34A1*/*SLC34A3*) to reduce renal reabsorption and it activates 1α-hydroxylase (15).

**Figure 1:**
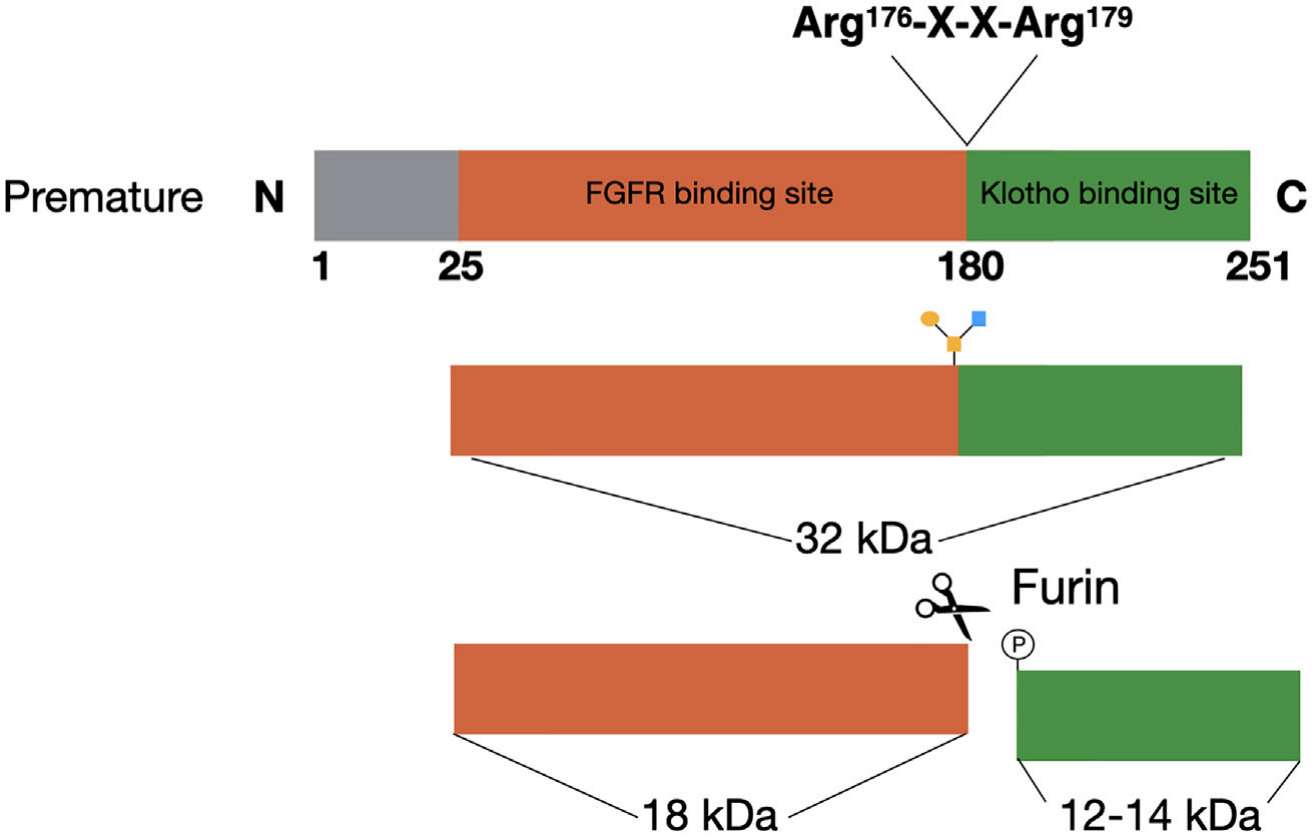
FGF23 processing. The premature FGF23 protein is a 251-amino acid long glycoprotein. In order to form the mature form of FGF23 the first 25 amino acids are removed through cleavage. FGF23 can be O-glycosylated at Thr^178^, which protects it from cleavage and results in active FGF23 in the circulation. The alternative is for FGF23 to be phosphorylated at Ser^180^. This results in cleavage at the consensus sequence (Arg^176^-X-X-Arg^179^) and inactive cleaved FGF23 in the circulation.

FGF23 is a member of the fibroblast growth factor (FGF) family, which has a plethora of functions. The FGFs are divided into subfamilies based on their structure and phylogenic analyses (16). FGF23 is a member of the FGF19 subfamily, which consists of FGF19, FGF21, and FGF23. The FGFs in this subfamily function as circulating hormones, and are therefore also called endocrine FGFs (17, 18). Several upstream regulators inhibit or induce the expression of FGF23 in the osteocytes and late osteoblasts, which will be extensively discussed in this review. The *FGF23* gene consists of three exons, that encode for a glycoprotein of 251 amino acids (19). This glycoprotein consists of three domains with the first 24 amino acids being the signal sequence, the middle part of 155 amino acids forming the core FGF homology domain and the last 72 amino acids comprising the C-terminal (20). The C-terminus of FGF23 is important for binding to klotho, while the N-terminus contains the FGFR binding domain (**Figure 1**) (17). The cleaved C-terminal domain competes with FGF23 for binding to klotho, and can therefore inhibit the formation of the FGF23/FGFR/Klotho complex (21). Studies performing overexpression of FGF23 in Chinese hamster ovary (CHO) cells have shown that the mature FGF23 protein lacks the signal peptide (22). Western blotting analyses of the recombinant media of these cells using an antibody against the C-terminus resulted in two products, one of ~30 kDa and a smaller one of ~10 kDa. This indicated that proteolytic processing is taking place before FGF23 is secreted (22).

FGF23 can be cleaved by subtilisin-like proprotein convertases at a consensus sequence (Arg^176^-X-X-Arg^179^), that is unique for FGF23 as it does not appear in other members of the FGF family (**Figure 1**) (22, 23). Alternatively, FGF23 can be O-glycosylated at Thr^178^ by polypeptide *N*-acetylgalactosaminyltransferase 3 (Gal-NAc-T3, encoded by *GalNT3*), which protects FGF23 from proteolytic cleavage (24). Phosphorylation of FGF23 at Ser^180^ *via* the secretory protein kinase family with sequence similarity-20 member C (FAM20C) prevents this O-glycosylation and thereby makes FGF23 more prone to cleavage by the proprotein convertase furin (24, 25). The balance between these processes determines the ratio between active intact FGF23 (iFGF23) and inactive cleaved FGF23 (cFGF23) that will be released into the circulation (26, 27).

Although still quite limited and with many more to discover, there are a number of factors known to regulate the expression and/or cleavage of FGF23. Below we provide a current overview of both classical and non-classical regulators of FGF23.

## Classical FGF23 Regulation

### Local FGF23 Regulators in Osteocytes

#### DMP1

**Figure 2:**
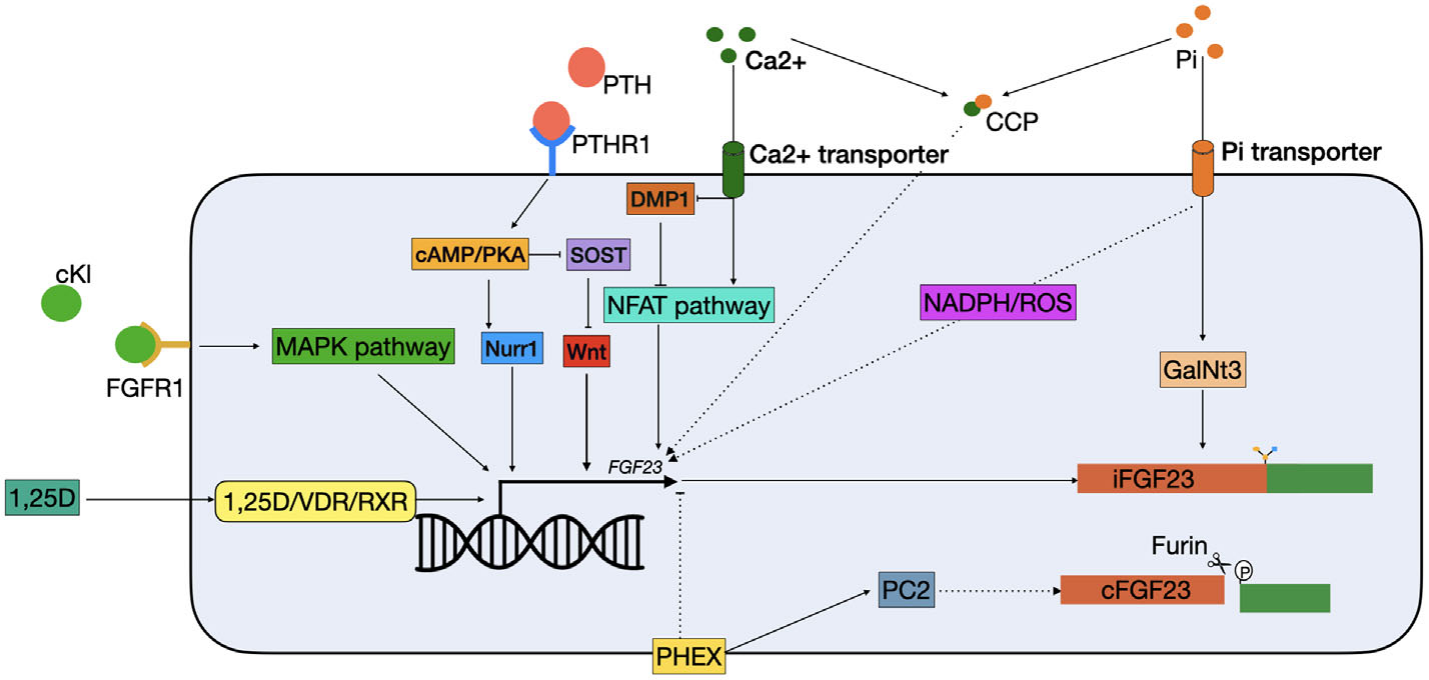
Schematic overview of classical FGF23 regulation. cKL binds to the FGFR1 and activates FGF23 transcription *via* the MAPK pathway. 1,25(OH)2D3 binds to the VDR, which heterodimerizes with the RXRs and can bind to the VDRE in the FGF23 promotor. PTH binds to the PTHR1 on the cell membrane, and activates the cAMP/PKA pathway. This has two effects: 1) NURR1 mRNA increases, resulting in increased FGF23 transcription; 2) inhibition of SOST, thereby indirectly stimulating *FGF23* transcription by releasing the suppression of the WNT pathway. Both Ca^2+^ and Pi can activate FGF23 transcription independently. Ca^2+^ enters the cell *via* a Ca^2+^ transporter. In the cells it inhibits DMP1, an inhibitor of the NFAT1 pathway, thus activating the NFAT pathway, leading to FGF23 transcription stimulation. Phosphate enters the cells through phosphate transporters. In the cell it stimulates FGF23 through the production of ROS through a yet unknown mechanism. Furthermore, it increases expression of GalNT3, resulting in protection from cleavage of the full-length FGF23 protein. Also, CCPs can increase FGF23 transcription but the mechanism remains unknown. Lastly, PHEX is able to inhibit FGF23 transcription through a yet unknown mechanism and interact with PC2 to increase FGF23 cleavage. 1,25D, 1,25-dihydroxyvitamin D_3_; Ca^2+^, calcium; cAMP, Cyclic adenosine 3′,5′-monophosphate; CCP, calciprotein particles; cFGF23, cleaved fibroblast growth factor 23; cKL, cleaved klotho; DMP1, dentin matrix protein 1; iFGF23, intact fibroblast growth factor 23; FGFR1, fibroblast growth receptor 1; GalNt3, polypeptide *N*-acetylgalactosaminyltransferase 3; MAPK, mitogen-activated protein kinase; NADPH, nicotinamide adenine dinucleotide phosphate; NFAT, nuclear factor of activated T cells; Nurr1, nuclear receptor related-1 protein; PC2, proprotein convertase; PHEX, phosphate regulating endopeptidase homolog X-linked; Pi, phosphate; PKA, protein kinase A; PTH, parathyroid hormone; PTHR1, parathyroid hormone receptor 1; ROS, reactive oxygen species; RXR, retinoid X receptor; SOST, sclerostin; VDR, vitamin D receptor.

Dentin matrix protein 1 (DMP1) is an extracellular matrix pro-peptide that is produced by osteocytes and a member of the small integrin binding ligand N-linked glycoprotein (SIBLING) family (28). DMP1 is a suppressor of FGF23 as inactivating mutations in DMP1 result in autosomal recessive hypophosphatemic rickets (ARHR), a disease where overproduction of FGF23 results in renal phosphate wasting, osteomalacia, and rickets (29). In mouse models of Dmp1 deficiency increased *Fgf23* transcription results from paracrine stimulation of Fgfr1, and downstream activation of the nuclear factor of activated T cells (Nfat) pathway (**Figure 2**) (30, 31). The FGF23 promotor contains an NFAT response element, which controls *FGF23* expression in response to calcium and inflammatory stimuli (32). Dussold et al. showed that *Nfat1* mRNA expression is increased in bone of chronic kidney disease (CKD) mice and that Dmp1 inhibits Nfat1 signaling that is activated in CKD, thereby preventing *Fgf23* transcription (**Figure 2**) (32). This only occurs in mice with early CKD but not in more advanced stages, suggesting that other stimuli such as elevated PTH and chronic inflammation may override this effect. In DMP1-treated mice with advanced CKD, there is a small decrease in circulating Fgf23, despite transcription levels remaining stable. This might indicate that DMP1 also has a post-translational effect on FGF23 (32). However, this study only measured the total levels of serum FGF23 and to gain more insight in how DMP1 might affect FGF23 processing, levels of both iFGF23 and cFGF23 should be measured. In conclusion, although it is clear that DMP1 has an inhibitory effect on *FGF23* transcription in CKD, studies are needed to describe its function in a healthy situation. Moreover, there are indications that DMP1 affects post-translational FGF23 modification, although it is unclear what the exact role of DMP1 in this process could be.

#### PHEX

Mutations in phosphate regulating endopeptidase homolog X-linked (PHEX) result in the most common form of chronic hypophosphatemia, named X-linked hypophosphatemia (XLH), by causing elevated levels of FGF23 (33). Hyp mice have an inactivating mutation of *Phex*, which mimics XLH in humans. They have a lower serum phosphate level due to high circulating levels of FGF23, which can be restored by a high phosphate diet (34). Moreover, dietary phosphate has been shown to improve bone growth and mineralization in hyp mouse, similar to what has been observed for patients with XLH (35).

PHEX is predominantly expressed by osteocytes and osteoblasts and encodes for an enzyme that degrades local SIBLINGs, in particular osteopontin (OPN) (36). Despite being an enzyme, it is thought that PHEX mostly suppresses the transcription of *FGF23* rather than its degradation, though the mechanism behind this is unknown (37, 38). There is some evidence that PHEX might indirectly play a role in FGF23 cleavage. Proprotein convertase (PC2), encoded by the proprotein convertase subtilisin/kexin-type-2 (PCSK2) gene, has been shown to be upregulated by PHEX, which can result in FGF23 cleavage *in vitro* (**Figure 2**) (39). Moreover, PC2 may promote the formation of PHEX-DMP1-integrin complexes that suppress FGF23 production when they are activated by neuroendocrine protein 7B2•PC2 (40). However, despite the fact that PC2 cleaves FGF23 *in vitro*, there is no evidence that the two proteins are in physical contact within the osteocyte (23).

PHEX mutations are X chromosome-linked, so females with a loss of function mutation in one PHEX allele should also have an unaffected second allele. This should result in functional proteins, but nevertheless women develop XLH as well (41). Preferential X chromosome inactivation in woman has been proposed as a mechanism to explain why XLH is not a recessive disease. However, a PCR analysis of 13 female XLH patients showed no significant difference in X inactivation patterns in peripheral blood cells. compared to healthy controls (42). Moreover, a case study of identical twins, of which only one was affected with XLH, also did not reveal a difference in X inactivation patterns as analyzed in peripheral blood cells (43). Nonetheless, it should be noted that X inactivation pattern in blood cells may not be representative for other tissues, especially in twins (44). It is therefore possible that in these cases there is preferential X inactivation in other tissues, such as bone, resulting in the XLH phenotype. All in all, even though mutations in *PHEX* have been established to disturb FGF23 regulation, the exact regulation of FGF23 by PHEX remains largely unknown to date.

### Circulating FGF23 Regulators

#### 1,25-Dihydroxyvitamin D_3_

*In vivo* studies show that C57Bl/6 mice injected with 1,25(OH)_2_D_3_ have significantly higher Fgf23 serum levels compared to vehicle injected mice. This increase was mostly attributed to increased *Fgf23* expression in the bone. When UMR106 osteosarcoma cells were treated with vitamin D, a significant upregulation of *Ffg23* expression was found after 4 h. Addition of actinomycin D, a gene transcription inhibitor, completely abolished the upregulation of *Fgf23*, thus showing that increased *Fgf23* expression by 1,25(OH)_2_D_3_ is due to a transcriptional effect (45). This effect is believed to be modulated *via* the vitamin D receptor (VDR) in osteocytes (46). This is also showed in patients with inactivating mutations in the *VDR* gene who have lower FGF23 serum levels (47). When 1,25(OH)_2_D_3_ binds to the VDR, it heterodimerizes with the retinoid X receptors (RXR), this complex can bind to the vitamin D response element (VDRE) in the promotor of target genes (**Figure 2**) (48). Treatment with 1,25(OH)_2_D_3_ in hypoparathyroid patients increases serum FGF23 and rapidly decreases serum phosphate, despite the fact that 1,25(OH)_2_D_3_ increases phosphate absorption in the gut (49). Moreover, Ito et al. demonstrated 1,25(OH)_2_D_3_-enhanced FGF23 promoter activity and mRNA expression in human chronic myelogenous leukemia K562 cells (46). Animal experiments have also shown that 1,25(OH)_2_D_3_ regulates Fgf23 levels. VDR null mice have low serum Fgf23 levels, which can be normalized with a rescue diet to normalize low phosphate levels and secondary hyperparathyroidism (50). However, thus far it has been unclear whether 1,25(OH)_2_D_3_ directly stimulates *FGF23* expression in osteocytes *via* the VDR, because osteocytes have proven difficult to isolate and culture (51). Yashiro et al. have shown that 1,25(OH)_2_D_3_ treatment results in increased *Fgf23* expression in the murine MC3T3-E1 osteocyte-like cell line. Knockdown of the VDR in osteocytes decreased *Fgf23* expression significantly, indicating that the response of Fgf23 to 1,25(OH)_2_D_3_ is primarily mediated through the VDRE in the promoter of the Fgf23 gene (52).

It is important to note that some of the work on Fgf23 regulation by 1,25(OH)_2_D_3_ is performed using mice and rats, which are both nocturnal species. Both mice and humans use UV-exposure as a 1,25(OH)_2_D_3_ source, but as mice are nocturnal, they have less opportunity to expose themselves to UV light. Additionally, both humans and mice use their diet as a source of vitamin D (53). Mutations in the *Vdr* or *Cyp27b1* genes in mice result in phenotypes similar to those seen in humans with these mutations (54–57). Even though vitamin D metabolism is different in mice and human, it does not preclude the use of mice as a suitable model to study the effect of 1,25(OH)_2_D_3_ on Fgf23.

#### PTH

Secondary hyperparathyroidism is a common complication of CKD triggered by hypocalcemia, hyperphosphatemia, and low vitamin D levels. These elevated PTH levels are usually found in combination with increased FGF23 levels (58). Moreover, it was found that PTH infusion resulted in increased FGF23 levels in healthy men, thus indicating that FGF23 is upregulated in patients with hyperparathyroidsism (59). PTH binds to the parathyroid hormone 1 receptor (PTH1R), which is expressed in numerous tissues including kidney and bone and activates several intracellular secondary messengers (**Figure 2**) (60, 61). In bone a major physiological role of PTH is to bind to the PTH1R on cells of the osteoblast lineage and enhancing the release of receptor activator of nuclear factor-κB ligand (RANKL), which then binds to its receptor RANK on osteoclast precursors, thereby enhancing osteoclast formation or activity (62). Data from osteoblast-like UMR-106 cells shows that PTH can also stimulate FGF23 production in osteoblasts by binding to the PTH1R and stimulating both the protein kinase A (PKA) and Wnt pathways (63). One study showed that PTH increases levels of *nuclear receptor related-1 protein (NURR1)* mRNA *via* the PKA pathway, and that overexpression of *NURR1* stimulates FGF23 in the absence of PTH in UMR-106 cells. Knockdown of *NURR1* prevented an increase in FGF23, indicating that NURR1 is essential for the stimulation of FGF23 by PTH in bone cells (64). Regulation of FGF23 by PTH *via* the Wnt pathway is through inhibition of sclerostin (SOST), a potent inhibitor of the Wnt pathway (65, 66). This effect is mediated by the PKA pathway. In adult Sabra rats, administration of Forskolin, a PKA inhibitor, resulted in decreased levels of *SOST* mRNA (62). Together, this indicates that activation of the PKA pathway by PTH both directly stimulates FGF23 transcription by increasing *NURR1* expression, as well as indirectly by inhibition of SOST (**Figure 2**).

#### α-Klotho

α-Klotho plays an important role in phosphate regulation by FGF23 as a co-receptor for FGFR1 in the kidney. Mice lacking α-klotho have an identical phenotype as the FGF23 KO mice, as FGF23 cannot fulfill its phosphaturic function in the kidney without α-klotho (8). Moreover, in a mouse model with a 70% reduction of α-klotho expression in the distal renal tubules, significantly higher levels of serum FGF23 were found (67). This indicates that the body attempts to compensate for lower levels of α-klotho expression, by upregulating FGF23. There are several isoforms of αklotho, of which the membrane-bound klotho is important for the interaction with FGF23 in the kidney. However, the circulating or cleaved form of klotho (cKL) has also been associated with FGF23 regulation (68). This relation between cKL and upregulation of FGF23 was discovered in 2008. An infant was diagnosed with severe hypophosphatemia, but no mutations were found *DMP1*, *PHEX*, *FGFR1*, or *FGF23*. She was found to have a chromosomal translocation (t9:13) in proximity to the *α-Klotho* locus. Analysis of the patient’s serum revealed elevated levels of cKL and FGF23, and resulted in the hypothesis that cKL can drive *FGF23* expression (69). In a mouse model mimicking this translocation, the treatment with cKl resulted in significantly higher levels of serum Fgf23 and a 150-fold increase of *Fgf23* mRNA in bone (70). Moreover, *c-fos* and *early growth response protein 1* (*Egr1*) were upregulated, both targets of the mitogen-activated protein kinase (MAPK) pathway, which is believed to be involved in activating *Fgf23* transcription (**Figure 2**) (70, 71). It is hypothesized that cKl plays a role in fine-tuning the levels of iFGF23, since activity of the FGFR1c was initiated by cKl in the presence of iFGF23 (70). To investigate this relationship, the osteoblastic cell line UMR-106 was treated with FGF23, cKl or a combination. While the combination resulted in upregulated levels of *EGR1* and *FGF23*, the single treatments were not capable of achieving this (72). Moreover, deletion of *FGFR1* by CRISPR/Cas9 also ablated this response (72). This implicates a relation between circulating cKl and circulating FGF23 levels. Administration of cKl to α-klotho null mice, resulted in upregulation of Fgf23 and the prevention of hyperphosphatemia and vascular calcification (72). This may represent a mechanism to prevent hyperphosphatemia when the circulating levels of FGF23 are not adequate. In this respect, cKl might also be of clinical interest, as it is able to prevent hyperphosphatemia even in the absence of functional α-klotho. It still needs to be determined whether cKl in normal circumstances is able to change levels of *FGF23* expression.

#### Phosphate

A major regulator of FGF23 levels is serum phosphate, which is critically maintained stable in the circulation by a combination of renal reabsorption, intestinal absorption, and bone resorption. Controlled feeding studies showed that high dietary phosphate intake results in higher levels of FGF23, while low intake results in a decrease (73–75). However, the mechanism behind phosphate-mediated FGF23 regulation remains unknown. Several studies measured phosphate levels in the serum before and after meals, but did not find a relation with diet (76, 77). Moreover, hyperphosphatemia in *glial cells missing homolog 2* (*Gcm2*) null mice and the addition of phosphate to osteoblast cultures did not result in increased *Fgf23* transcripts, a result that was also found in other studies (78–80). However, a study by Takashi et al. showed that mice fed a high phosphate diet have increased levels of serum Fgf23 compared to mice on a low phosphate diet (71). They found in UMR106 cells that this was not caused by the upregulation of *Fgf23* transcription, but rather by increased expression of *GalNt3*, which results in a higher level of active iFGF23 in the circulation (71). Nonetheless, other studies do show that high extracellular phosphate results in enhanced *FGF23* transcription (81, 82). When 4 mM phosphate was added to IDG-SW3 cells, this resulted in a 38-fold increase in *Fgf23* expression. Interestingly, this effect was not found in the presence of 10 mM phosphate (82). Another study reported that phosphate enhances *FGF23* transcription in UMR-106 cells. In addition, they also found that phosphate resulted in increased production of reactive oxygen species (ROS). When an inhibitor of nicotinamide adenine dinucleotide phosphate (NADPH) oxidase was used, not only the production of ROS diminishes, but also *FGF23* transcription (**Figure 2**) (81). There are no studies that show *in vivo* that ROS production is correlated with FGF23 production but it is known that patients with renal failure have increased ROS production (83). Studies assessing whether production of ROS is correlated with the risk of adverse events in the presence of high FGF23 levels are therefore warranted. It is interesting that FGF23 is mostly known as a phosphaturic hormone, but that the exact mechanism by which phosphate regulates it (production, cleavage, secretion) remains largely unclear. This underlines the importance for more research about FGF23 regulation by phosphate in both healthy individuals and patients with abnormal phosphate levels.

#### Calcium

A study in rats showed that increased *Fgf23* transcription in response to high dietary phosphate is only possible when serum calcium levels are also high (84). This is in agreement with another study showing that it is not the increase of phosphate itself that increases *Fgf23* transcription, but rather the formation of calciprotein particles (CCPs) (85). CCPs are colloidal nanoparticles that consist of calcium and phosphate circulating in the blood and are potentially harmful as they contribute to vascular calcification. UMR106 cells treated with synthesized CCPs showed increased *FGF23* mRNA (85). Moreover, in C57BL/6 mice it was shown that the amount of plasma CCPs were increased after a high dietary phosphate load. In this study they also showed that the high phosphate diet also resulted in more circulating FGF23 and increased levels of mRNA *Fgf23* in the bone (85). The authors hypothesize that this mechanism might constitute a negative feedback loop, since activating FGF23 results in lower levels of serum phosphate, which in turn will decrease the amount of harmful CCPs that can be formed (**Figure 2**). There is also evidence that calcium itself is able to upregulate *FGF23* expression. In CKD, serum FGF23 levels are elevated due to hyperphosphatemia, and dietary phosphate restriction and phosphate binders are being used to keep FGF23 levels under control. Interestingly, studies showed that calcium-containing phosphate binders do not result in a decrease of serum FGF23 levels, while calcium-free phosphate binders do decrease FGF23 levels (86). Indicating that calcium itself might also modulate FGF23 in the absence of phosphate. Moreover, *Cyp27b1^−/−^* mice fed a high calcium diet had higher levels of serum FGF23. Besides, adding calcium to the culture medium of MC3T3-E1 cells resulted in higher *Fgf23* expression (87). A recent study in B6 wild-type mice showed that an injection of calcium led to increased levels of circulating Fgf23 in 6 h. When an NFAT-inhibitor, 11R-VIVIT, was used, this decrease was partially inhibited. DMP1 overexpression in B6 DMP^TG^ mice also partially prevented this increase (32). Together, this indicates that at least part of the increase of FGF23 levels can be explained by a direct induction by calcium through DMP1 inhibition and the NFAT-pathway (**Figure 2**). However, since inhibiting the NFAT pathway did not completely eradicate the FGF23 induction by calcium, research is needed to study whether the remaining effect is by the formation of CCPs or a tertiary mechanism.

## Non-Classical FGF23 Regulation

### Iron Deficiency

**Figure 3:**
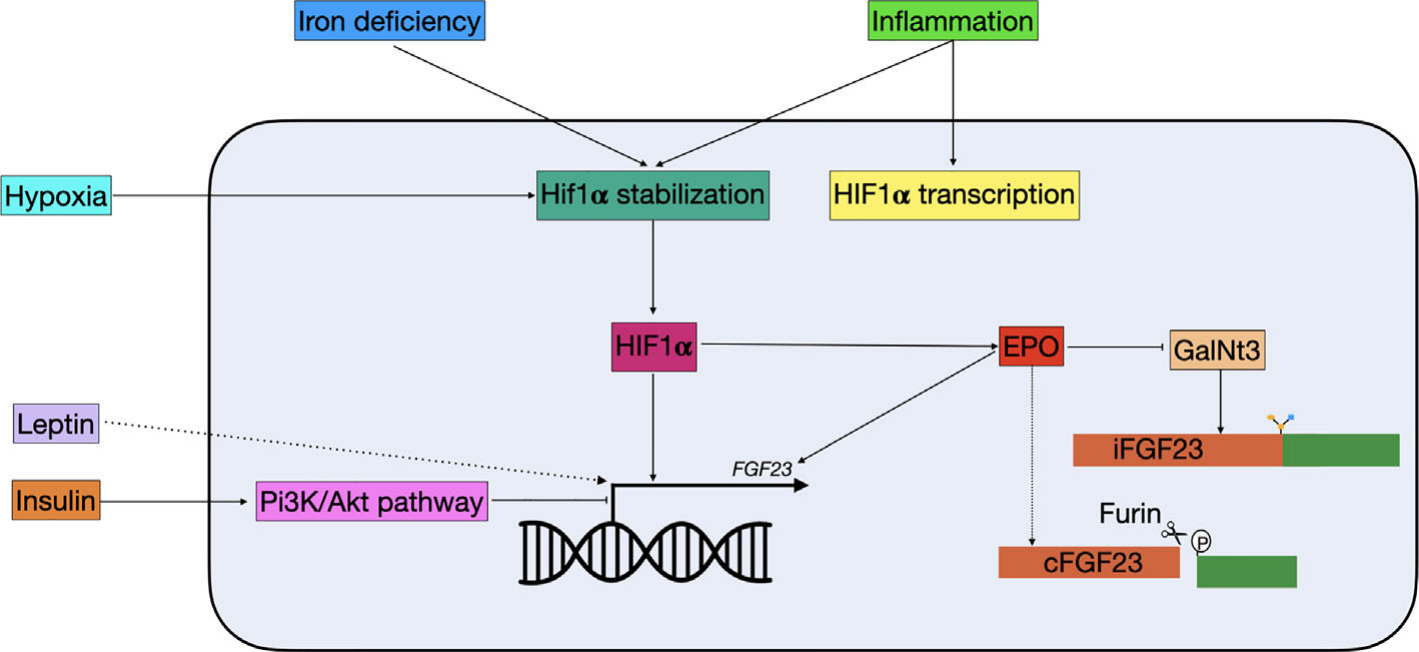
Schematic overview of non-classical FGF23 regulation. Hypoxia, iron deficiency, and inflammation all result in Hif1α stabilization. Hif1α, which can be stimulated by inflammation, binds to the HRE in the *FGF23* promotor and stimulate expression. Hif1α can also indirectly regulate FGF23: 1) it stimulates EPO, which directly stimulates FGF23 transcription and 2) it inhibits GalNt3, resulting in increased FGF23 cleavage. Insulin is able to inhibit FGF23 transcription through the Pi3K/Akt pathway, while leptin stimulates FGF23 transcription through a yet unknown mechanism. cFGF23, cleaved fibroblast growth factor 23; EPO, erythropoietin; iFGF23, intact fibroblast growth factor 23; GalNt3, polypeptide *N*-acetylgalactosaminyltransferase 3; HIF1α, hypoxia inducible factor 1 α; PI3K/Akt, phosphatidylinositol 3-kinase/protein kinase B.

In 1982 a link between iron and hypophosphatemia was first described in patients receiving repeated intravenous therapy with saccharated ferric oxide (88). Later, clinical observations in ADHR patients suggested that there is a correlation between iron and FGF23 (89). In ADHR patients with low serum iron levels, higher levels of both iFGF and cFGF23 were found, while low serum iron levels in healthy individuals only result in elevated cFGF23 but not iFGF23 (90). A recent study in a mouse model of CDK showed that anemia is the primary driver of increased Fgf23 in CDK and that this can be rescued by treating the mice with erythropoiesis stimulating agents (91). These observations suggested that there is a role for iron in FGF23 regulation. In a study where wild-type mice were treated with hepcidin, a reactant that causes inflammation-induced iron deficiency, *Fgf23* transcription was increased along with elevated levels of cFgf23, but not iFgf23 (92). A similar result was found in mice treated with low-iron diets and iron chelators, where mRNA levels of *Fgf23* were increased, but levels of iFgf23 were only slightly elevated (**Figure 3**). Interestingly, the levels of iFgf23 significantly increased in CKD mice when iron deficiency was induced (92). This indicates that iron deficiency increases transcription and cleavage of FGF23 simultaneously, and that elevated circulating levels of iFGF23 are only present in situations where FGF23 cleavage is impaired, such as in CKD and ADHR patients. Paradoxically, when otherwise healthy women with iron deficiency were treated with ferric carboxymaltose, half of them developed hypophosphatemia (93). This was attributed to an acute increase in iFGF23 levels, while cFGF23 levels were decreased. When these women were treated with iron dextran the levels of cFGF23 decreased as expected but without the spike in iFGF23 (93). The mechanism by which ferric carboxymaltose increases iFGF23 levels is unknown. It could be possible that it promotes O-glycosylation of FGF23 and thereby protects it from cleavage. It should be noted that considering the fact that iron dextran administration did not cause this acute rise in iFGF23, it could be caused by the sugar molecule rather than the iron supplementation. Nevertheless, clinicians should be aware of the effect that iron supplementation can have on mineral homeostasis.

### Inflammation

In clinical studies, it has been shown that increased inflammation markers correlate with increased serum FGF23 levels, however, the balance between its production and cleavage is maintained, as in iron deficiency (**Figure 3**) (94, 95). Moreover, a recent study using a mouse model of CKD has shown that production of the pro-inflammatory protein IL-1β is the driving stimulus for upregulation of *Fgf23* expression in early CKD. The usage of a neutralizing antibody to IL-1β blocked the expression of Fgf23 (96). Treatment with inflammatory factors in IDG-SW3 cells also resulted in increased *Fgf23* mRNA levels, but not iFgf23 in conditioned medium (97). In mice that were pretreated with a furin inhibitor, serum iFGF23 levels increased rapidly following treatment with inflammatory factors, further indicating an essential role for furin in the cleavage of intact FGF23 (92). Part of the increased *Fgf23* mRNA may be mediated through an enhancer region 16 kDa upstream of the *Fgf23* gene (98). Although deletion of the enhancer in a mouse model did result in lower levels of *Fgf23* mRNA upon stimulation with LPS, TNF-α, and IL-1β, levels of circulating iFGF23 did not change (85).

In multiple inflammatory conditions, there is a role for nuclear factor kappa-light-chain-enhancer of activated B cells (NF-κB) (99). Several studies have shown that NF-κB is involved in increased *FGF23* transcription, *via* Orai1 activation (100, 101). It is therefore possible that part of the increased transcription of *FGF23* can be explained by NF-κB activation.

### Erythropoietin

Both inflammation and functional iron deficiency stimulate *Fgf23* transcription indirectly through the production of erythropoietin (EPO) (102). Injection of recombinant human EPO in mice resulted in increased *Fgf23* mRNA and cFgf23, but only marginally increased iFgf23, indicating that production and cleavage of FGF23 are directly linked, as observed in inflammation and iron deficiency (102, 103). It is currently unknown why both transcription and cleavage are upregulated during iron deficiency and inflammation. One hypothesis is that cFGF23 has a role in a hitherto undiscovered endocrine feedback loop involved in iron homeostasis and erythropoiesis, without affecting phosphate and calcium homeostasis (104). In a mouse model of acute blood loss, increased levels of cFgf23 were found while *GalNt3* mRNA was decreased (105). This might be a mechanism by which changes in oxygen tension and erythropoiesis promote cFGF23 levels, without disturbing the levels of biological active iFGF23 (**Figure 3**) (104). Most experiments involving EPO are relatively short term, e.g., several hours or days of EPO treatment, while in a clinical setting EPO is given for months or even years. Therefore, research has been done in Tg6 mice, a model constitutively overexpressing human EPO (106). In these mice both iFgf23 and cFgf23 were increased at 6–8 weeks compared to their wild-type littermates (107). Moreover, these mice showed signs of disturbed mineral metabolism, including reduced trabecular bone mineral density, low renal αklotho, urinary calcium wasting, and reduced expression of renal and intestinal phosphate transporters (107). Together this indicates that long-term increases in EPO are able to disturb mineral metabolism, and that the relation between bone mineralization and EPO therapy should be investigated in a clinical setting.

### HIF1α

Iron deficiency and inflammation are both able to alter *FGF23* transcription *via* hypoxia inducible factor 1 α (HIF1α). HIF1α is stabilized in case of hypoxia or iron deficiency and activates downstream pathways (92). Moreover, HIF1α is believed to be involved in altered FGF23 cleavage seen in inflammation and iron deficiency. When HIF1α inhibitors were given simultaneously with the IL-1β treatment, iFGF23 levels were elevated in wild-type mice, indicating that HIF1α is directly involved in increased FGF23 cleavage caused by inflammation (92). However, HIF1α may not directly stimulate FGF23, but instead act through EPO. In a study in rats it was shown that treatment with human recombinant EPO resulted in a higher increase in cFGF23 compared to the ones treated with a HIF propyl-hydroxylase (PH) inhibitor, which stabilizes HIF (105). In rats that were pre-treated with anti-EPO antibodies, the increased cFGF caused by HIF PH inhibitor treatment was completely abolished, indicating that EPO may be the mediator in the increase of cFGF23 in response to HIF stabilization (108). Nonetheless there are some studies that suggest that HIF1α stabilization directly influences *FGF23* transcription (**Figure 3**). Fluorescent-tagged antibodies for both HIF1α and FGF23 co-localize in perivascular cells in resected tumors of patients with TIO and chromatin immunoprecipitation assays showed that HIF1α might bind to a HIF-binding site within the FGF23 promotor (109).

For a deeper understanding of the interplay between iron, inflammation, EPO and HIF1α more research to FGF23 biology is needed. The cFGF23 fragments that are increased during iron deficiency and inflammation, do not seem to represent the classical role of FGF23 in maintaining mineral homeostasis. It is therefore important to study how exactly these processes are able to increase both FGF23 transcription and cleavage.

### Insulin and Diabetes

In clinical studies diabetes was found to be associated with higher serum levels of FGF23 (110, 111). Both diabetes type 1 and 2 are associated with high levels of inflammation, which is positively correlated with high levels of FGF23 (112, 113). It is therefore possible that the relation between diabetes and high FGF23 levels is mostly explained by inflammation. However, Bär et al. showed in UMR106 cells that insulin treatment is able to suppress *FGF23* gene expression in the absence of inflammatory factors (114). Most of the downstream effects of insulin are mediated through the PI3K/PKB/Akt signaling pathway, and inhibition of this pathway or its downstream transcription factor, FOXO1, ablated the effect of insulin on FGF23 expression (**Figure 3**) (114, 115). Moreover, in a diabetic mouse model, where use of streptozocin ablates insulin production through pancreatic β-cell destruction, a spike in serum Fgf23 was seen, which could be prevented by giving the mice daily injections of insulin (114). Finally, the authors looked at a correlation between FGF23 levels and fasted insulin concentration in healthy human volunteers. They found that the baseline insulin concentration was inversely correlated with FGF23 levels. When the volunteers were subjected to a glucose challenge, the increase in insulin was also inversely correlated with FGF23 levels (114). Together these results indicate that insulin is an inhibitor of FGF23, at least partially independent of the inflammation associated with diabetes.

### Leptin

Leptin (encoded by *LEP*) is a hormone mainly produced by adipocytes and secreted in response to a change in body energy stores (116). Leptin deficient *ob/ob* mice overexpress *25(OH)D*, *1,25(OH)_2_D_3_*, and *1α-hydroxylase* and a lower expression of *Fgf23* (117, 118). Administration of leptin to these mice corrects for the overexpression of *25(OH)D, 1,25(OH)_2_D_3_*, and *1α-hydroxylase* (117). However, leptin does not inhibit 1α-hydroxylase in primary cell cultures, suggesting the involvement of another factor (118). Moreover, when leptin is administrated to *ob/ob* mice this results in an increase of serum Fgf23 levels, while decreasing serum phosphate, calcium and 1,25(OH)_2_D_3_, an effect that was not found in leptin receptor-deficient *db/db* mice (118). The authors also investigated whether administrating Fgf23 to *ob/ob* mice had similar effects as leptin, which resulted in a normalization of *1α-hydroxylase* expression (118). Together this indicates that leptin is able to directly stimulate *Fgf23* expression in *ob/ob* mice (**Figure 3**). In a clinical study, higher levels of leptin have also been associated with higher levels of FGF23. However, this association was not found to be significant when leptin was included in a multivariate-adjusted model (119). Even though there are some strong indications that leptin indeed stimulates FGF23, the mechanism behind it is unknown. Additional work should reveal the interaction between these hormones in order to add leptin to the definitive list of FGF23 regulators.

## Discussion

Since the discovery of FGF23 in the year 2000, much knowledge has been gathered about the upstream regulators of this hormone. In this review, we distinguish the classical regulation of FGF23 involved in phosphate metabolism and the non-classical regulation affected by inflammation and iron or insulin levels. Irregularity in these processes underlie disorders in bone and mineral metabolism and insights in FGF23 regulation should provide insights with potential therapeutic consequences. Even though insights in FGF23 biology are advancing, many of the mechanisms behind it are still unclear.

### Considerations When Studying FGF23 Regulation

FGF23 is primarily produced by osteocytes, which are challenging cells to study (120). Osteocytes are terminally differentiated cells and are hard to isolate in sufficient quantities. In the past years several cell lines have been developed from mouse osteocytes, which are also used in a large portion of the studies described in this review (121). However, these cell models have their limitations, as they have to be modified in order to dedifferentiate and proliferate, while osteocytes *in vivo* do not (122–124). Much of the research discussed in this review was performed in osteoblast cell lines (e.g., MC3T-E1), but it still remains controversial whether osteoblasts are truly a source of FGF23, as it is mostly described as a late osteocyte marker (123, 125, 126). Moreover, the osteosarcoma cell line UMR106 is often used, but as this is a cancerous cell line, FGF23 regulation might be altered/deranged (127). Because all these cell lines differ from the osteocytes found *in vivo* it is hard to state with certainty that mechanism found in these cell lines are representative of the situation in humans or animals.

Secondly, osteocytes are embedded in the mineralized bone matrix, and therefore might behave differently than when they are cultured on a flat surface. Research progress regarding 3D culture models might be a solution for this problem. One recent study showed that primary murine osteoblasts gain a more osteocyte-like pattern when cultured in 3D and dedifferentiated to a more osteoblast-like pattern when placed back in 2D (128). For OCY454 cells it has been shown that culturing in a 3D scaffold resulted in a more robust increase in *Fgf23* transcription following PTH treatment (124). Another study showed that primary human osteocytes had a higher expression of *SOST* and *FGF23* when they were cultured in 3D in hypoxic conditions (129). Together these results indicate that optimizing the culture conditions for osteocytes is important to study FGF23 as it would function *in vivo*.

The most promising option to study FGF23 regulation might be the culture of human bone chips, as they will mimic the *in vivo* situation more closely (130). However, limited availability and donor variation makes it difficult to produce consistent results. There is a long way to go before we can study human FGF23 biology in its natural human environment, but with the currently available models it will be possible to gain valuable insights in FGF23 regulation.

### Biological Role for cFGF23

The intent of classical FGF23 regulation is unambiguous: maintaining phosphate homeostasis in the body. For non-classical FGF23 regulation this is not as straightforward, as inflammation or iron deficiency will not only result in higher *FGF23* expression but also in increased cleavage. It is currently unclear whether this is a protective mechanism to maintain mineral homeostasis when HIF1α is stabilized or that the cFGF23 fragments have a yet undefined function. Based on findings in mice where cFGF23 inhibited FGF23 signaling, it can be hypothesized that cFGF23 acts as a competitive inhibitor to the FGF-receptor (21). Still, healthy humans and animals with iron deficiency often have normal serum phosphate levels (90, 131, 132). This makes it unlikely that cFGF23 would have a role in FGF23 inhibition in healthy individuals, as they would be expected to present with hyperphosphatemia. It is also possible that cFGF23 plays a role in a yet to be discovered feedback loop in maintaining iron homeostasis and erythropoiesis. Further research needs to be done to identify the precise effect of cFGF23 fragments, as they seem to have no role in classical FGF23 regulation but might have unique properties instead.

### FGF23 Cleavage Machinery

The discovery of genetic mutations affecting FGF23 cleavage elegantly showed that the intracellular FGF23 cleavage machinery is essential for its functioning. Although it is clear that proteases such as furin are involved in cleaving FGF23, and that phosphorylation by FAM20C and O-glycosylation by Gal-NAc-T3 regulate this process, the exact mechanisms of FGF23 cleavage are unknown (24). The mechanisms by which FAM20C is transcriptionally and functionally regulated remains to be elucidated, though studies suggest that changes in Gal-NAc-T3 and FAM20C activity or transcription seem to be regulated by iron, iron deficiency and elevated serum phosphate (24, 133, 134). It is likely that FGF23 cleavage is far more complicated than this but whether and how other upstream regulators affect cleavage and which other proteins might be involved remain unanswered questions. For deeper understanding of FGF23 regulation it is therefore important to study how this process exactly works and what effect upstream regulators have.

### Emerging Regulators

Apart from the factors described here, some other upstream regulators of FGF23 have emerged in the past years and undoubtedly there are more to be discovered. Factors that were found to cause an upregulation of *FGF23* expression are glycerol-3-phosphate, cytoskeleton reorganization, NF-κB/Orai1 signaling, aldosterone, and cytosolic calcium activity (100, 101, 135, 136). These factors are mostly described in single *in vitro* studies or small clinical studies, and it is therefore not yet fully understood why and how they affect *FGF23* transcription. This emphasizes how much there is still to uncover about FGF23 and all of its potential upstream regulators. Additionally, recent advances in RNA sequencing and bioinformatics have revealed hitherto unknown roles of circular RNAs, long non-coding RNAs (lncRNAs), Piwi-interacting (pi)RNAs, and micro (mi)RNAs in various signaling pathways (137, 138). However, the role of these non-coding RNAs in osteocyte function is still unexplored (139). Studies to the role these non-coding RNAs in osteocyte function and FGF23 signaling, could therefore unravel a whole new class of FGF23 regulators.

### Therapeutic Therapies for FGF23-Mediated Pathologies

Conventional treatment for children with XLH consists of 1,25(OH)_2_D_3_ and phosphate supplementation, which results in improved growth and less rachitic deformities. However, outcomes are variable and the treatment comes with adverse effects such as hypercalcemic hyperparathyroidism and nephrocalcinosis (140). A new promising therapy is the monoclonal FGF23 antibody burosumab, which has been shown to normalize phosphate levels in both adults and children. Burosumab is currently FDA approved for the treatment of XLH and future studies need to show whether this drug can also be used in other FGF23-mediated pathologies, such as TIO (140, 141). Besides these established therapies, experimental treatments included the use of growth hormone, which seemed to improve growth during the therapy but did not result in a significant difference in adult height (142). Some new treatment options are currently being tested in hyp mice. These include 1) the use of hexa-D-arginine which increases the expression of 7B2, which indirectly suppresses *FGF23* expression (39) and and 2) methods to block the FGFR in hyp mouse, which results in improved growth (143). Many of the current therapies focus on blocking FGF23 activity or managing the effect of high FGF23 levels. This underlines the importance of developing therapies that will target the upstream regulators of FGF23 directly, instead of managing the downstream consequences.

## Conclusion

Advances over the past 20 years have provided knowledge regarding several regulators of FGF23 both involved in mineral metabolism and possible alternative processes. Moreover, it has become clear that FGF23 transcription and cleavage are independently regulated processes. The way upstream regulators affect these actions is not always clear. It is also yet to be discovered why the osteocyte would increase FGF23 production in response to some of these factors, only to immediately cleave them. Future research answering these questions might not only provide valuable insights in the molecular mechanisms regulating FGF23, but may also be of use to develop new therapeutic strategies for patients with FGF23-associated disorders.

## Author Contributions

DR performed the literature research, wrote the manuscript and designed the figures. DR, MZ, and BE revised the manuscript. All authors contributed to the article and approved the submitted version.

## Funding

DR is supported by a grant from Health Holland (PhosphoNorm; LSHM18029).

## Conflict of Interest

The authors declare that the research was conducted without any commercial or financial relationships that may be regarded as a potential conflict of interest.
